# Magnetic Resonance Imaging in Tauopathy Animal Models

**DOI:** 10.3389/fnagi.2021.791679

**Published:** 2022-01-25

**Authors:** Ruiqing Ni

**Affiliations:** ^1^Institute for Biomedical Engineering, ETH Zurich and University of Zurich, Zurich, Switzerland; ^2^Institute for Regenerative Medicine, University of Zurich, Zurich, Switzerland

**Keywords:** magnetic resonance imaging (MRI), tau, animal model, FTD (frontotemporal dementia), Alzheimer’s disease, neurodegenaration

## Abstract

The microtubule-associated protein tau plays an important role in tauopathic diseases such as Alzheimer’s disease and primary tauopathies such as progressive supranuclear palsy and corticobasal degeneration. Tauopathy animal models, such as transgenic, knock-in mouse and rat models, recapitulating tauopathy have facilitated the understanding of disease mechanisms. Aberrant accumulation of hyperphosphorylated tau contributes to synaptic deficits, neuroinflammation, and neurodegeneration, leading to cognitive impairment in animal models. Recent advances in molecular imaging using positron emission tomography (PET) and magnetic resonance imaging (MRI) have provided valuable insights into the time course of disease pathophysiology in tauopathy animal models. High-field MRI has been applied for *in vivo* imaging in animal models of tauopathy, including diffusion tensor imaging for white matter integrity, arterial spin labeling for cerebral blood flow, resting-state functional MRI for functional connectivity, volumetric MRI for neurodegeneration, and MR spectroscopy. In addition, MR contrast agents for non-invasive imaging of tau have been developed recently. Many preclinical MRI indicators offer excellent translational value and provide a blueprint for clinical MRI in the brains of patients with tauopathies. In this review, we summarized the recent advances in using MRI to visualize the pathophysiology of tauopathy in small animals. We discussed the outstanding challenges in brain imaging using MRI in small animals and propose a future outlook for visualizing tau-related alterations in the brains of animal models.

## Introduction

Six microtubule-associated protein tau (MAPT) isoforms are expressed in the adult human brain and are further categorized into 4-repeat (4R) and 3-repeat (3R) species (Lee et al., 2001). Tauopathy diseases include Alzheimer’s disease (AD) and primary tauopathies such as progressive supranuclear palsy (PSP), corticobasal degeneration (CBD), and frontotemporal dementia (FTD) with Parkinsonism linked to chromosome 17, and Pick’s disease (Ballatore et al., 2007). Primary tauopathies are pathologically characterized by the aggregation of hyperphosphorylated tau protein into neurofibrillary tangles, neuropil threads, and argentophilic glial inclusions (Lee et al., 2001). Thus, tau has been an important target in therapeutic development for AD and primary tauopathies, with several immunotherapies, antisense oligonucleotides, and aggregation inhibitors under clinical trials (DeVos et al., 2017; Congdon and Sigurdsson, 2018; Boxer et al., 2019, 2020; Ayalon et al., 2021; Grossman, 2021; Mullard, 2021; Novak et al., 2021). Animal models recapitulating tauopathy have facilitated the understanding of disease mechanisms and the development of treatment strategies (Albert et al., 2019; Roberts et al., 2020; Ayalon et al., 2021), including transgenic mouse lines P301S (PS19), EC-tau, P301L (JNPL3, rTg4510, pR5), rTg21221 (Lewis et al., 2000; Ramsden et al., 2005; Santacruz et al., 2005; Yoshiyama et al., 2007; Hoover et al., 2010; de Calignon et al., 2012) and rat models (Filipcik et al., 2012). In addition, knock-out hTau (Andorfer et al., 2003) and knock-in mouse models (Hashimoto et al., 2019; Saito et al., 2019; Hosokawa et al., 2021) have been recently developed. Animal models exhibit tau accumulation, neuroinflammation, synaptic dysfunction, brain regional atrophy, and cognitive impairment to different extents (Götz et al., 2018; Ishikawa et al., 2018; Ising et al., 2019). Magnetic resonance imaging (MRI) has been widely used to non-invasively probe the tissue changes associated with cerebral tau pathology in patients with AD and FTD (Du et al., 2006; Boxer et al., 2020; Young et al., 2021). Regional atrophy assessed by T_2_-weighted MRI, white matter integrity assessed by diffusion tensor imaging (DTI), and cerebral perfusion measured by arterial spin labeling (ASL) have emerged as potential biomarkers in AD and FTD. Recent advances in MRI and contrast agents (Wahsner et al., 2019) have provided valuable insights into the time course of disease pathophysiology in tau animal models, including tau, neuroinflammation, and structural and functional alterations.

## Tau Imaging

**FIGURE 1:**
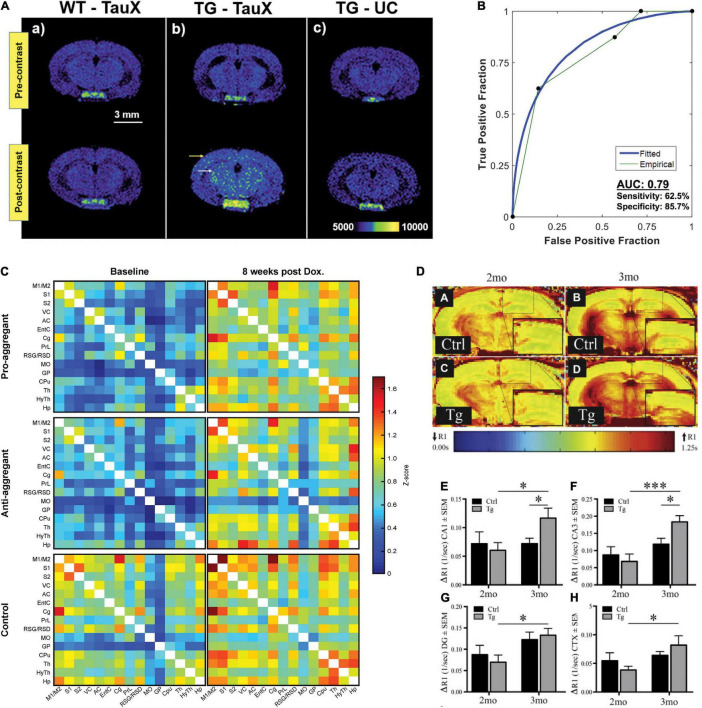
Tau accumulation and functional imaging in tauopathy mouse models. **(A,B)** Tau imaging. **(A)** T_1_-weighted spin-echo images demonstrate signal enhancement in delayed post-contrast scans of transgenic P301S mice treated with TauX but not in age-matched controls. Wild-type animals showed no signal enhancement 4 days after the administration of TauX. Transgenic animals show high enhancement in the cortical (yellow arrow) and hippocampal regions (white arrow) 4 days after the administration of TauX. Transgenic animals showed no signal enhancement 4 days after the injection of untargeted contrast. Scale bar represents 3 mm. All animals are shown on the same color bar scale. **(B)** Receiver operating characteristic curve demonstrating TauX accuracy in identifying early age transgenic animals. Area under the curve is calculated using the empirical curve. Sensitivity describes the true positive rate for transgenic mice given TauX, while specificity is the true negative rate for wild-type mice given TauX and transgenic mice receiving untargeted gadolinium nanoparticle contrast. Reproduced from Badachhape et al. (2020) with permission from Elsevier. **(C)** Functional connectivity matrices of transgenic rTg4510 and wild-type mice at baseline and after 8weeks of treatment with doxycycline. The pro- and anti-aggregant mice clearly show matrices of lower *z* score values at baseline (left column). Upon 8weeks of doxycycline treatment (right column), the *z* score values of the transgenic animals closely approximate those of the control group. Reproduced from Green et al. (2019) with permission from Springer Nature. **(D–H)** MEMRI-R1 detects early signs of neuronal dysfunction in transgenic rTg4510 mice before the onset of cognitive deficits. **(D, A–D)** Representative R1 map of **(A)** 2-month littermate control, **(B)** 3-month littermate control, **(C)** 2-month rTg4510 mouse (Tg), and **(D)** 3-month rTg4510 mouse (Tg). Quantification of ΔR1 values in **(E)** CA1, **(F)** CA3, **(G)** dentate gyrus, and **(H)** superior medial cortex. All values are mean ± SEM, *n* = at least 4. ***p < 0.001, *p < 0.05. Reproduced from Fontaine et al. (2017) with permission from Elsevier. CA1, cornu ammonis 1; CA3, cornu ammonis 3; CTX, cortex; DG, dentate gyrus; MEMRI, manganese-enhanced magnetic resonance imaging.

Tau is located intracellularly and is subject to posttranslational modifications, including phosphorylation, acetylation, ubiquitylation, and truncation. Tau aggregates into nanofibrils with cofactors (Goedert et al., 1996) and displays typical sigmoidal kinetics of nucleation-dependent protein aggregation (Chakraborty et al., 2021). The smaller aggregates, tau oligomers, are considered more neurotoxic than the neurofibrillary tangle, induce synaptic and mitochondrial dysfunction, and impair memory functions in animal models (Lasagna-Reeves et al., 2011). Tau imaging has been even more challenging than amyloid imaging due to the intracellular location and size of tau aggregates, as well as the different tau isoforms (Villemagne et al., 2015). Positron emission tomography (PET) imaging of tau in tauopathy mouse models using various tau-targeted radioligands has been established (Maruyama et al., 2013; Brendel et al., 2016; Ono et al., 2017; Ishikawa et al., 2018; Ni et al., 2018; Tagai et al., 2020; Chaney et al., 2021; Cao et al., 2022). In addition, fluorescence imaging, two-photon microscopy, and photoacoustic imaging methods have been developed for *in vivo* mapping of tau accumulation in animal models (Wu et al., 2018; Calvo-Rodriguez et al., 2019; Ni et al., 2020a; Vagenknecht et al., 2021). To date, a few MRI tau imaging studies have been reported assisted with contrast agents (Table 1). Two MRI contrast agents have been reported for detecting tau in animal models *in vivo*. Yanagisawa et al. (2018) reported that the [^19^F]buta-1,3-diene derivative Shiga-X35 allowed the detection of tau aggregates in the forebrain region of 8–9-month-old female rTg4510 mice compared with wild-type mice at 7 T MRI. The detection was verified by colocalization with anti-phosphorylated tau antibody AT8-positive tau deposits. Badachhape et al. (2020) and Parekh et al. (2021) demonstrated tau imaging using the DNA aptamer-targeted liposomal-Gd nanoparticle TauX, which binds to the surface of hyperphosphorylative cells, for T_1_-weighted spin echo MRI in PS19 mice (Figure 1A). Increased TauX-enhanced post-contrast MR signal enhancement was detected at 2 months of age in PS19 mice (which showed tauopathy 4–6 months later) compared with wild-type mice, with an accuracy of approximately 0.8 (Figure 1B).

**TABLE 1:** Summary of MRI in animal models of tauopathy.

Target	MRI sequence	Animal model	References
WM	DTI	pR5 mice	Massalimova et al., 2021; Soni et al., 2021
		rTg4510 mice	Sahara et al., 2014; Wells et al., 2015b; Colgan et al., 2016; Holmes et al., 2016
		TauRD/ΔK280 mice	Green et al., 2019
		Thy-Tau22	Degiorgis et al., 2020
		JNPL3 mice	Nishioka et al., 2019
		Tg601 mice	Hara et al., 2017
Tau	^19^F Shiga-X35	rTg4510 mice	Yanagisawa et al., 2018
	TauX, T_1_w-SE	PS19 mice	Badachhape et al., 2020; Parekh et al., 2021
Atrophy	T_2_	rTg4510 mice	Xie et al., 2010; Yang et al., 2011; Wells et al., 2015b; Holmes et al., 2016; Ishikawa et al., 2018; Ni et al., 2018; Ma et al., 2019; Barron et al., 2020; Park et al., 2020; Wang et al., 2020
		hTau mice	Hashimoto et al., 2019
		EC-tau mice	Fung et al., 2020
		pR5 mice	Kindler et al., 2021
		rTg21221 mice	Musi et al., 2018
		PS19 mice	Wu et al., 2019; Takeuchi et al., 2020; Lee et al., 2021
		R962-hTau rats	Malcolm et al., 2019
Texture	T_2_*w MRTA, T_2_*w	rTg4510 mice	Colgan et al., 2017; O’Callaghan et al., 2017
	QSM		O’Callaghan et al., 2017
Neurochemical profiles	^1^H MRS	rTg4510 mice	Yang et al., 2011; Kim et al., 2017
	^1^H, ^13^C MRS	pR5 mice	Nilsen et al., 2013
	CEST	Tau4RΔK (Tau) mice	Chen et al., 2021
		rTg4510 mice	Wells et al., 2015b; Holmes et al., 2016
		Tau4RΔK (Tau) mice	Chen et al., 2020
		hTau mice	Lauretti et al., 2017
		PS19 mice	Crescenzi et al., 2014, 2017
Optic nerve	T_2_	rTg4510 mice	Harrison et al., 2019
Fe, Ca	SWI	pR5 mice	Ni et al., 2020b
CVR	ASL	rTg4510 mice	Wells et al., 2015a
CMRO_2_	TRUST. PC	Tau4RΔK (Tau) mice	Wei et al., 2021
CBF	ASL	rTg4510 mice	Wells et al., 2015b; Holmes et al., 2016; Park et al., 2020
		PS19 mice	Park et al., 2020
		Tau.P301L mice	Govaerts et al., 2019
		pR5 mice	Kindler et al., 2021
		Tau4RΔK mice	Wei et al., 2021
BOLD	rs-fMRI	Thy-Tau22 mice	Degiorgis et al., 2020
		TauRD/ΔK280 mice	Green et al., 2019
		hTau.P301L mice	Detrez et al., 2020
	Task-based fMRI	rTg4510 mice	Nahavandi et al., 2017
Glymphatic system	DCE-MRI	rTg4510 mice	Harrison et al., 2020
Synaptic function	MEMRI	rTg4510 mice	Pautler et al., 2003; Perez et al., 2013; Majid et al., 2014; Fontaine et al., 2017; Bachstetter et al., 2020; Koren et al., 2020
		JNPL3 mice	Bertrand et al., 2013
		Tau-KO mice	Lopes et al., 2016
		Wtau-Tg mice	Kimura et al., 2007

*ASL, arterial spin labeling; BOLD, blood-oxygenation-level-dependent; CBF, cerebral blood flow; CE, contrast enhanced; CEST, chemical exchange saturation transfer imaging; CMRO_2_, cerebral metabolic rate of oxygen; CVR, cerebral vascular response; DCE, dynamic contrast enhanced; DTI, diffusion tensor imaging; fMRI, functional magnetic resonance imaging; ME, manganese enhanced; MRI, magnetic resonance imaging; MRS, magnetic resonance spectroscopy; MRTA, magnetic resonance texture analysis; PC, phase contrast; QSM, quantitative susceptibility mapping; rs, resting state; SE, spin echo; SWI, susceptibility-weighted imaging; TRUST, T_2_ relaxation under spin tagging; WM, white matter; w, weighted.*

## Functional Imaging

Hyperneuronal activity has been shown to enhance tau secretion and exacerbate tau pathology in several tauopathy mouse models, including rTg4510, EC-Tau (Wu et al., 2016), Thy-Tau22 (Gomez-Murcia et al., 2020), and the TAU58/2 lines (Przybyla et al., 2020). Pathological tau accumulates mainly in excitatory neurons rather than in inhibitory neurons, leading to neuronal network dysfunction and neural circuit impairment (Busche et al., 2008; Fu et al., 2019). Functional imaging techniques, such as manganese-enhanced MRI (MEMRI), ASL, resting-state functional MRI (rs-fMRI), and contrast-enhanced fMRI, have been widely used to probe brain functional alterations in small animals.

### Resting-State Functional MRI and Task-Based fMRI

Blood-oxygen-level-dependent (BOLD) signals measured by rs-fMRI are widely used for non-invasive mapping of brain function between neural activity and its accompanying hemodynamics. Recent studies have shown that the spreading of misfolded tau follows a disease-specific region-dependent pattern in the brain not only in the anatomically connected regions but also in the functionally connected regions in patients with AD (Franzmeier et al., 2020; Vogel et al., 2021) and with FTD (Kim et al., 2020; Spinelli et al., 2021; Young et al., 2021). The default mode network (DMN) is a set of network nodes consisting of the medial prefrontal cortex, the posterior cingulate/precuneus, inferior parietal lobe, lateral temporal cortex, and hippocampal formation (Buckner and DiNicola, 2019). In patients with primary tauopathy, DMN regions are affected at an early stage (Zhou et al., 2010; Lee et al., 2014). Few fMRI studies have been reported thus far in tau mouse models (Table 1). As these studies are performed in different mouse lines, a direct comparison of the results is infeasible. Green et al. (2019) showed that functional networks were impaired by elevated tau accumulation in TauRD/ΔK280 mice compared with wild-type mice and were reversible by doxycycline treatment to regulate soluble tau for 8weeks (under 1.5% isoflurane during rs-fMRI) (Figure 1C). Degiorgis et al. (2020) demonstrated that there was hyperactivated functional connectivity in the hippocampus, amygdala, and isocortical areas in Thy-Tau22 mice at an early stage (5 months of age) compared with wild-type mice using rs-fMRI (maintained under medetomidine sedation during fMRI), which preceded the occurrence of memory impairment. A different observation was reported by Detrez et al. (2020), who found that progressive tau aggregation did not alter the functional brain network connectivity in hTau.P301L mice at 7 months of age after seeding with K18 tau at 3 months of age (under 0.5% isoflurane and medetomidine during fMRI) at 7 T MRI. Using task-based fMRI at 9.4 T, Nahavandi et al. (2017) reported differences in the visual processing pathway and a stronger midbrain BOLD response to visual stimulation in rTg4510 mice than in wild-type mice at 7.5 months of age. Interpretation of BOLD signals is challenged by their dependence on multiple factors, such as baseline physiological state, breathing, animal handling, ventilation, and anesthesia scheme (Grandjean et al., 2014a; Paasonen et al., 2018). In addition, variation in the fMRI signal might partly stem from technical factors, fluctuations, spatial localization, non-linearities, task, pulse sequence used, and data analysis approaches.

### Manganese-Enhanced MRI

Manganese-enhanced MRI (MEMRI) is a sensitive *in vivo* neuroimaging method that detects the neuronal activity-based transport of Mn^2+^ into active neurons (Silva and Bock, 2008). Several *in vivo* MEMRI studies have demonstrated axonal transport deficits in rTg4510 (Perez et al., 2013; Majid et al., 2014; Fontaine et al., 2017; Bachstetter et al., 2020; Koren et al., 2020), Tau-KO (Lopes et al., 2016), JNPL3 (Bertrand et al., 2013), and Wtau-Tg mice (Kimura et al., 2007) compared with control mice (Table 1). Using MEMRI at 7 T, Fontaine et al. (2017) showed elevated changes in tissue R1 relaxation rates (Δ*R*1) after the administration of Mn^2+^, indicating early neuronal dysfunction at 3 months of age in rTg4510 mice before the onset of cognitive deficits (Figures 1D–H). However, concerns regarding the neurotoxicity of Mn^2+^ hinder the wide application of this method.

### Arterial Spin Labeling

**FIGURE 2:**
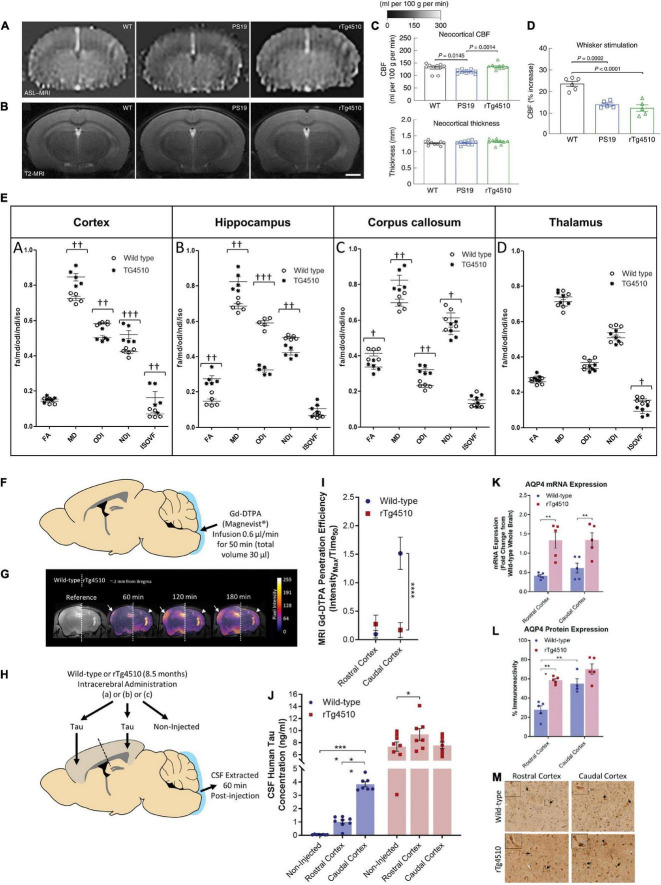
Reduced cerebral blood flow, white matter integrity, glymphatic inflow, and clearance of Tau in rTg4510 mice. **(A)**, Neocortical CBF, as assessed by ASL–MRI [representative images on the left, quantification in **(C)**], shows no reduction in 2–3-month-old rTg4510 mice and a small reduction in 2–3-month-old PS19 mice compared with age-matched WT mice. *N* = 10 mice per group; one-way ANOVA with Tukey’s test for multiple comparisons. **(B)** Neocortical thickness, as assessed bilaterally in T_2_-weighted MRI [images (representative images on the left, quantification in **(C)**] at the level of the somatosensory cortex (-1.22—1.70mm from bregma), is comparable in PS19, rTg4510, and WT mice. Scale bar, 1mm. *N* = 10 mice per group. **(D)** The increases in CBF induced in the whisker barrel cortex by mechanical stimulation of facial whiskers (*N* = 5 mice per group; one-way ANOVA with Tukey’s test) were markedly attenuated in both PS19 and rTg4510 mice compared with WT mice. Reproduced from Park et al. (2020) with permission from Springer Nature. **(E)** Neurite orientation dispersion and density imaging for white matter integrity assessment. Region-of-interest quantification of fractional anisotropy, mean diffusivity (× 10^– 9^ m^2^/s), orientation dispersion index, neurite density index, and isotropic volume fraction for each animal based on distinct anatomical regions. **(A)** Cortex, **(B)** hippocampus, **(C)** corpus callosum, and **(D)** thalamus († = *p* < 0.05, †† = *p* < 0.01, ††† = *p* < 0.001). Reproduced from Colgan et al. (2016) with permission from Elsevier. **(F)** Schematic illustrating infusion of Gd-DTPA into the cisterna magna of the mouse for quantification of glymphatic inflow in the brain. **(G)** Representative pseudocolor scaled coronal (approximately -2 mm from bregma) images of the (*left*) wild-type and (*right*) rTg4510 mouse brain, highlighting the difference in the extent of contrast agent infiltration into the caudal cortex [designated by white arrows (wild-type) and arrowheads (rTg4510)] over time. This difference is further exemplified through the calculated Gd-DTPA penetration efficiency data shown in **(I)**. **(H)** Schematic illustrating brain homogenate injection experiments in which tau-containing brain homogenate was injected into either the rostral or caudal cortex of wild-type and rTg4510 mice, and CSF was extracted from the cisterna magna 60 min later. **(J)** Tau concentration of CSF samples extracted from experiments shown schematically in d demonstrating reduced clearance from the caudal cortex of rTg4510 mice compared with wild-type animals. Raw data and mean ± SEM between animals shown in d, and best-fit value and associated 95% CI of sigmoidal fitting of data shown in c. *n* = 5–8 per group. Statistical significance is denoted by asterisks: **p < 0.01, ****p < 0.0001. AQP4 expression and polarization in rTg4510 mice. Quantification of **(K)** mRNA and **(L)** protein expression of AQP4 in the rostral and caudal cortex of wild-type and rTg4510 mice, demonstrating upregulation in rTg4510 mice compared with wild-type controls. **(M)** Representative example images of brain tissue from wild-type and rTg4510 mice immunohistochemically stained for AQP4. Arrows indicate examples of immune-positive blood vessels in each image, which are shown at greater magnification in insets. Reproduced from Harrison et al. (2020) with permission from the Oxford press. AQP4, aquaporin 4; CSF, cerebrospinal fluid; FA, fractional anisotropy; Gd-DTPA, gadolinium diethylenetriaminepentaacetic acid; IsoVF, isotropic volume fraction; MD; mean diffusivity, NDI, neurite density index; NODDI, neurite orientation dispersion and density imaging; ODI, orientation dispersion index; WT, wild-type.

Arterial spin labeling MRI has been widely used in the clinical setting as well as in preclinical imaging in animal models. ASL MRI has demonstrated different spatial distributions of hypoperfusion in patients with FTD compared with AD (Du et al., 2006; Verfaillie et al., 2015; Meeter et al., 2017). ASL uses magnetically labeled blood water and allows the direct quantification of absolute cerebral blood flow (CBF) and cerebrovascular reactivity of the whole brain regions. In regions with very short or very long arterial transit times, the accuracy of ASL measurement is compromised. Previous studies on ASL measures of CBF in tauopathy animal models have yielded inconsistent results (Table 1). Park et al. (2020) showed that at 2–3 months of age, rTg4510 mice had comparable resting CBF, attenuated CBF response to whisker stimulation, and no cortical thinning compared with wild-type mice. Reduced resting CBF and CBF responses to whisker stimulation in the neocortex and the hippocampus, along with reduced cortical thickness, were detected at 7–8 months of age compared with wild-type mice (Figures 2A–D; Park et al., 2020). In the PS19 mice, an early impaired resting CBF and CBF response to whisker stimulation at 2–3 months of age was detected in the absence of cortical thinning compared with wild-type mice (Figures 2A–D). Moreover, tau induces postsynaptic protein PSD95-neuronal nitric oxide uncoupling and neurovascular dysfunction in rTg4510 and PS19 mice in a neurodegeneration-independent manner (Park et al., 2020). In contrast, a study by Wells et al. (2015b) and Holmes et al. (2016) reported elevated levels of CBF in the cortex, hippocampus, and thalamus in 7.5–9.5-month-old rTg4510 mice compared with wild-type mice. Another recent study by Govaerts et al. (2019) showed that the regional CBF levels are comparable between Tau.P301L mice and wild-type mice at 3, 6, and 12 months of age. Kindler et al. (2021) showed a comparable cortical and hippocampal CBF between pR5 mice and wild-type mice at 10 and 18 months of age. Wei et al. (2021) reported a reduced cerebral metabolic rate of oxygen while preserving CBF in Tau4RΔK (Tau) mice at 12 months of age compared with wild-type mice by using T_2_ relaxation under spin tagging, phase contrast, and ASL MRI. For cerebral vascular response quantification, pulsed ASL and pseudocontinuous ASL methods have been utilized. Using a hypercapnia (5% carbon dioxide)-challenged pulsed ASL vasoreactivity paradigm, Wells et al. (2015a) showed increased vascular responses to hypercapnia conditions in rTg4510 mice compared with wild-type mice at approximately 8 months of age. It is noted that information regarding the amount of carbon dioxide in exhaled air was not provided in this article. The varying results regarding the influence of tau on CBF (increasing, preserved, or reducing) require further investigation.

## Structural MRI

### Volumetric MRI

MRI investigations have revealed disease-specific patterns of gray and white matter atrophy in patients with PSP, CBD, Pick’s disease, and variants of FTD and AD, providing valuable tools for differential diagnosis (Boxer et al., 2006; Jabbari et al., 2020; Ulugut Erkoyun et al., 2020; Vogel et al., 2021; Young et al., 2021). In animal models of tauopathy, histological studies have demonstrated the deposition of neurofibrillary tangle pathology, particularly in the cortex and hippocampus, accompanied by regional atrophy. Both *in vivo* and *ex vivo* MRI studies have been performed to assess structural alterations. Ma et al. (2019) compared the results from *in vivo* and *ex vivo* volumetric MRI in rTg4510 mice and reported comparable readouts of brain atrophy compared with wild-type mice. MRI studies in animal models of tauopathies have revealed distinctive neuroimaging features and patterns of brain regional atrophy in rTg4510, hTau, EC-Tau, rTg21221, and PS19 mice as well as in R962-hTau rats (Table 1). Gray matter atrophy, cortical thinning, hippocampal atrophy, and ventricle enlargement that result from neurodegeneration have been reported in several MR studies in the rTg4510 tau mouse model compared with wild-type mice (Yang et al., 2011; Wells et al., 2015b; Holmes et al., 2016, 2017; Ishikawa et al., 2018; Ni et al., 2018; Ma et al., 2019; Barron et al., 2020; Park et al., 2020). In addition, Colgan et al. (2017) and O’Callaghan et al. (2017) reported tau-related tissue textural alterations using T_2_*-weighted MR textural analysis and T_2_*-weighted quantitative susceptibility mapping (QSM) in rTg4510 mice. In addition to the aforementioned MRI studies, optic nerve thinning and degeneration in the neurosensory retina have been reported in rTg4510 mice (Harrison et al., 2019).

In comparison with wild-type mice, hTau mice showed predominantly cortical thinning and rather spared hippocampi (Andorfer et al., 2003; Hashimoto et al., 2019). For P301S (PS19) mice, Yoshiyama et al. (2007) showed atrophy in the hippocampus and entorhinal cortex at 9–12 months of age and that atrophy was further present in the amygdala and neocortex at later stages (Wu et al., 2019; Takeuchi et al., 2020; Lee et al., 2021). Takeuchi et al. (2020) demonstrated that tauopathy and MRI-assessed brain atrophy and cognitive impairment in PS19 mice can be reversed by nasal vaccine delivery. In EC-TAU mice, Fung et al. (2020) showed that regional hippocampal atrophy (based on tensor-based morphometry analysis) compared with age-matched wild-type mice was associated with tau pathology preceding overt cell death. In rTg21221 mice that overproduced non-aggregating wild-type human tau but lacked neurofibrillary tangle accumulation, mainly ventricle enlargement was observed compared with wild-type mice (Wegmann et al., 2015; Musi et al., 2018). Malcolm et al. (2019) characterized recently developed R962-hTau rats and reported ventricular dilation and hippocampal atrophy in this model.

### Diffusion Imaging

Diffusion imaging is based on the tissue water diffusion rate: DTI enables indirect measurement of the degree of anisotropy and structural orientation (Le Bihan et al., 2001), while diffusion kurtosis imaging (DKI) reports non-Gaussian water diffusion (Jensen and Helpern, 2010). DTI has been widely used to assess white matter integrity alterations non-invasively both in animal models and in the clinical setting (Mori and Zhang, 2006). The DTI scalars reflect various alterations: fractional anisotropy (FA) infers microstructural integrity, axonal diameter, and density of crossing fibers; radial diffusivity (RD) is the diffusivity perpendicular to axonal fibers and reflects myelin abnormalities; mean diffusivity (MD) is the average mobility of water molecules related to the white matter tissue microstructure; and axial diffusivity (AD) is the magnitude of diffusion parallel to fiber tracts associated with axonal pathologies (Assaf and Pasternak, 2008). Several DTI studies in tauopathy models have been reported, including rTg4510 (Sahara et al., 2014; Wells et al., 2015b; Holmes et al., 2016), Thy-Tau22 (Degiorgis et al., 2020), pR5 (Soni et al., 2021), Tg601 (Hara et al., 2017), JNPL3 (Nishioka et al., 2019), and TauRD/ΔK280 mice (Green et al., 2019), with varying results. Sahara et al. (2014) reported an age-dependent decrease in FA in the corpus callosum and anterior commissure in rTg4510 mice at 8 months of age, increased RD, and unaltered MD and AD in the corpus callosum. Colgan et al. (2016) found a lower FA and a higher MD in the corpus callosum of rTg4510 mice of 8.5 months of age compared with wild-type mice. Using neurite orientation dispersion and density imaging (NODDI) model, Colgan et al. (2016) showed that neurite density index (NDI) was increased in the cortex (with high tau load) but decreased in the hippocampus and corpus callosum of rTg4510 mice compared with wild-type mice, isotropic volume fraction (IsoVF) was increased in the cortex but decreased in the thalamus (a region void of tau) of rTg4510 mice compared with wild-type mice, and orientation dispersion index (ODI) was found to be reduced in the cortex and hippocampus but increased in the corpus callosum of rTg4510 mice compared with wild-type mice (Figure 2E). FA was increased in the hippocampus but reduced in the corpus callosum of rTg4510 mice compared with wild-type mice (Figure 2E; Colgan et al., 2016). Furthermore, the NDI readout correlated with the level of tau (PG-5 antibody staining) in the gray matter of the rTg4510 mouse brain. Wells et al. (2015b) demonstrated unaltered FA, MD, and AD and an increased RD in the corpus callosum and increased FA and MD in the gray matter (cortex and hippocampus) in rTg4510 mice compared with wild-type mice. Massalimova et al. (2021) showed decreased FA and increased MD, RD, and AD in the corpus callosum and decreased FA in the gray matter of the hippocampus in the pR5 line at 8.5 months of age compared with wild-type mice. Degiorgis et al. (2020) demonstrated a significant decrease in FA and fiber density in Thy-Tau22 mice at 5 months of age compared with wild-type mice. A DTI study in patients with FTD reported reduced FA values in the anterior corpus callosum, anterior and descending cingulum, and uncinate fiber tracts (Zhang et al., 2009; Torso et al., 2021). In comparison, in patients with AD, reduced FA was reported in the descending cingulum, posterior and anterior cingulum, and uncinate fiber tracts, correlating with the increased tau distribution assessed by PET (Kantarci et al., 2017; Jacobs et al., 2018; Sintini et al., 2019; Carlson et al., 2021). Thus, DTI may facilitate the differential diagnosis of FTD and AD in the clinical setting (Torso et al., 2021).

## MRI for Neurochemical Profiles

### MR Spectroscopy

Magnetic resonance spectroscopy (MRS) is a highly sensitive MR method for characterizing neurochemical alterations *in vivo* using the infusion of substrates labeled with magnetic isotopes (Zhu and Barker, 2011). However, MRS has not been translated to clinical usage, where fast, simple, and reliable measurement is essential. A few MRS studies have been reported thus far on tauopathy animal models. Yang et al. (2011) revealed an increase in the hippocampal myoinositol to total creatine ratios (mIns/tCr, representing gliosis) in rTg4510 mice at 5 and 8 months of age compared with wild-type mice using ^1^H MRS at 9.4 T. Kim et al. (2017) demonstrated more pronounced neurochemical alterations in the olfactory bulbs than in the hippocampus in rTg4510 mice by using ^1^H MRS. Nilsen et al. (2013) showed that glutamate metabolism is impaired in P301L (pR5) mice compared with wild-type mice by using ^1^H and ^13^C MRS.

### Chemical Exchange Saturation Transfer MRI

Molecular MRI based on chemical exchange saturation transfer (CEST) is a highly sensitive method that has enabled the detection of changes in the uptake of glucose, glutamate, and creatine without additional hardware (Nilsen et al., 2013; Mamoune et al., 2021). Recent advances in CEST MRI provide information on oxygen metabolism and tissue metabolite levels in the brain, with an increased translational value compared with MRS (Oz et al., 2014). Clinical CEST MRI has been reported using 3 and 7 T MRI (Jones et al., 2018; van Zijl and Knutsson, 2019; van Zijl et al., 2021). Protein-based amide proton transfer-weighted (APTw) CEST MRI has been reported in patients with mild cognitive impairment (Heo et al., 2019; Zhou et al., 2019; Zhang et al., 2020). Glucose CEST has been applied in several tau animal models (Table 1). Lauretti et al. (2017) showed reductions in brain glucose uptake and synaptic function, increased tau accumulation and phosphorylation, and memory impairments in hTau mice compared with control mice. Similar observations of reduced glucose uptake were reported in rTg4510 mice compared with wild-type mice (Wells et al., 2015b; Holmes et al., 2016). Chen et al. (2020) demonstrated reduced glucose uptake in a Tau4RΔK (Tau) mouse model by using on-resonance variable delay multiple pulse (onVDMP) MRI with improved labeling efficiency and sensitivity (Xu et al., 2019). Crescenzi et al. (2014, 2017) demonstrated that there is a reduction in glutamate levels in the hippocampus of PS19 mice compared with wild-type mice, as measured by longitudinal glutamate CEST. Glutamate reduction was found to be associated with the level of synapse loss Crescenzi et al., 2014, 2017). Using creatine CEST, Chen et al. (2021) recently detected a reduction in the cerebral creatine level in Tau4RΔK (Tau) mice compared with wild-type mice.

## Discussion

There has been a rapid development in MRI technology in recent years, particularly in high-field MR scanners. For clinical application in humans, 7 and 10.5T MRIs have been reported (Ehman et al., 2017; Ladd et al., 2018). For small animal imaging, 7, 9.4, 11.7, 16, and up to 21.1 T high-field MRI has been utilized in the laboratory (Schepkin et al., 2010; Miyaoka and Lehnert, 2020; Ni, 2021), providing insights into the function and pathophysiology of the brain. Higher magnetic fields substantially increase the sensitivity and signal-to-noise ratio for MRI, although the tissue heating and non-uniformity of the radio-frequency field might affect the image quality. In addition, hybrid imaging systems such as PET-MRI have been increasingly used in preclinical imaging research for complementary molecular and anatomical information (Musafargani et al., 2018; Stortz et al., 2018).

### Difference Among Animal Models

Different tauopathy mouse/rat models show distinct tempo-spatial patterns of pathological features, including tau deposits and regional atrophy (Jankowsky and Zheng, 2017; Götz et al., 2018). Tau accumulates in the entorhinal cortex, forebrain, and hippocampus of rTg4510 mice and mainly in the brainstem and spinal cord of the PS19 model (Clavaguera et al., 2009; Maruyama et al., 2013; Wegmann et al., 2019). In rTg4510 mice, atrophy was observed both in the cortex and in the hippocampus, while in hTau mice, cortical thinning was observed while the hippocampus was spared (Andorfer et al., 2003; Santacruz et al., 2005; Ni et al., 2018). Systematic approaches are needed for the direct comparison of datasets from different model systems (Oblak et al., 2020; Sukoff Rizzo et al., 2020).

### Methodology and Reproducibility Considerations

Diverging results have been reported in the aforementioned functional MRI studies in tauopathy mice. The various anesthetic regimens used in these studies add to the complexity in interpreting the results. Anesthesia usage brings the benefit of excellent motion control while at the cost of potential interference with measures, particularly in fMRI-related experiments. The regional connectivity in the mouse brain has been shown to be influenced by the different anesthesia protocols utilized (Wu et al., 2017; van Alst et al., 2019). In addition to the isoflurane or low-dose isoflurane + medetomidine sedation that has been used in the aforementioned studies, ketamine and xylazine mixtures have also been reportedly used to achieve stable states in mice or rats (Grandjean et al., 2014b,^2020^; Shim et al., 2018; Mandino et al., 2020). A recent systematic review summarized the influence of anesthetics, doses, and timing on fMRI results in rodents (Steiner et al., 2021). Thus, standardization of pipelines is important for better interpretation of the results from fMRI studies. Physiological parameters such as heartbeat, breathing rate, and mouse/rat body temperature are routinely included in all *in vivo* animal studies. In addition, three physiological parameters, namely, blood oxygenation (arterial blood pressure of oxygen), ventilation (arterial partial pressure of carbon dioxide), and arterial blood pressure, can influence BOLD fMRI readouts in various study designs (Steiner et al., 2020) and are thus recommended for inclusion in the monitoring of animal status. Moreover, Chen et al. (2019) reported real-time monitoring and adaptive modulation of the brain hemodynamic system to further facilitate fMRI experiments.

### Time Course of MRI Biomarkers in rTg4510 Mice

**FIGURE 3:**
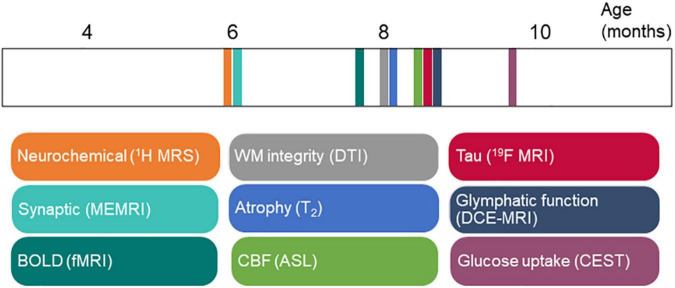
Time course of magnetic resonance imaging (MRI) biomarker changes in rTg4510 mice in Table 1. ASL, arterial spin labeling; BOLD, blood-oxygenation-level-dependent; CBF, cerebral blood flow; CEST, chemical exchange saturation transfer imaging; DCE, dynamic contrast enhanced; DTI, diffusion tensor imaging; fMRI, functional magnetic resonance imaging; ME, manganese enhanced; MRI: magnetic resonance imaging; MRS, magnetic resonance spectroscopy; WM, white matter.

The rTg4510 mouse model is one of the most widely used tauopathy animal models (Santacruz et al., 2005). rTg4510 mice express high levels of mutant tau (approximately 13 times compared with the levels of endogenous murine tau) and develop neurofibrillary tangles (from 4 months of age), neuroinflammation, neuronal loss, and behavioral impairments with increasing age (Ramsden et al., 2005). A recent study showed that factors other than hTau overexpression contributed to the tauopathy-like phenotype in this model (Gamache et al., 2019). Although no MR angiography has been performed in rTg4510 mice, Harrison et al. (2020) investigated the paravascular fluid (glymphatic) movement in this model: impaired glymphatic flow and clearance of tau in rTg4510 mice compared with wild-type mice were detected by using dynamic contrast-enhanced MRI assisted with Gd-DTPA (Figures 2F–M). In this study, we summarized the findings from the aforementioned MRI studies regarding the time course of events in rTg4510 mice (Figure 3). Following known tau accumulation from 2 months of age (Santacruz et al., 2005), early alterations in synaptic function and neurochemical profiles were observed. The changes in BOLD fMRI, CBF, white matter integrity, and regional atrophy appeared at a later stage. Most of the reported MRI studies are cross-sectional in rTg4510 mice of different age groups. More MRI studies with longitudinal and multiparameter designs are needed to elucidate the time course of these events.

### Translational Value

Longitudinal multimodal MRI, such as ASL, DTI, and rs-fMRI, has shown great potential as a diagnostic biomarker in FTD (Jiskoot et al., 2019) and for monitoring clinical trials (Staffaroni et al., 2019), providing added value to patients. Preclinical studies in animal models recapitulating human tauopathy provide an opportunity to study disease mechanisms and for extrapolation to human studies. In animal models, the measurements of structural alterations such as brain regional atrophy and white matter integrity yielded more homogenous results compared with the functional changes. As discussed in the “Functional Imaging” section, functional imaging studies using rs-fMRI and ASL MRI for CBF measurement have resulted in different observations in tau animal models. With the presence of such a discrepancy, special attention and further improvement of the protocol and standardization will be required if functional connectivity and CBF are to be used as evaluation readouts for treatment studies in animal models. Several recent studies have utilized awake fMRI to investigate the dysfunction of neural circuits in rodent disease models (Stenroos et al., 2018; Bergmann et al., 2020; Tsurugizawa et al., 2020; Dinh et al., 2021). Such a method will greatly increase the translational potential of the results from rodents to humans.

In summary, MRI studies in tauopathy animal models have improved our understanding of the roles of tau and the progression of pathophysiology and facilitated the evaluation of treatment studies targeting tau. Further MRI studies are needed to further characterize the functional, structural, and molecular alterations in various tauopathy animal models.

## Author Contributions

The author confirms being the sole contributor of this work and has approved it for publication.

## Conflict of Interest

The author declares that the research was conducted in the absence of any commercial or financial relationships that could be construed as a potential conflict of interest.

## Publisher’s Note

All claims expressed in this article are solely those of the authors and do not necessarily represent those of their affiliated organizations, or those of the publisher, the editors and the reviewers. Any product that may be evaluated in this article, or claim that may be made by its manufacturer, is not guaranteed or endorsed by the publisher.
